# Rapid response to fast viral evolution using AlphaFold 3-assisted topological deep learning

**DOI:** 10.1093/ve/veaf026

**Published:** 2025-04-29

**Authors:** JunJie Wee, Guo-Wei Wei

**Affiliations:** Department of Mathematics, Michigan State University, East Lansing, MI 48824, United States; Department of Mathematics, Michigan State University, East Lansing, MI 48824, United States; Department of Biochemistry and Molecular Biology, Michigan State University, East Lansing, MI 48824, United States; Department of Electrical and Computer Engineering, Michigan State University, East Lansing, MI 48824, United States

**Keywords:** topological deep learning, deep mutational scanning, AlphaFold 3, protein–protein interactions, SARS-CoV-2 variants

## Abstract

The fast evolution of SARS-CoV-2 and other infectious viruses poses a grand challenge to the rapid response in terms of viral tracking, diagnostics, and design and manufacture of monoclonal antibodies (mAbs) and vaccines, which are both time-consuming and costly. This underscores the need for efficient computational approaches. Recent advancements, like topological deep learning (TDL), have introduced powerful tools for forecasting emerging dominant variants, yet they require deep mutational scanning (DMS) of viral surface proteins and associated three-dimensional (3D) protein–protein interaction (PPI) complex structures. We propose an AlphaFold 3 (AF3)-assisted multi-task topological Laplacian (MT-TopLap) strategy to address this need. MT-TopLap combines deep learning with TDA models, such as persistent Laplacians (PL) to extract detailed topological and geometric characteristics of PPIs, thereby enhancing the prediction of DMS and binding free energy (BFE) changes upon virus mutations. Validation with four experimental DMS datasets of SARS-CoV-2 spike receptor-binding domain (RBD) and the human angiotensin-converting enzyme-2 (ACE2) complexes indicates that our AF3-assisted MT-TopLap strategy maintains robust performance, with only an average 1.1% decrease in Pearson correlation coefficients (PCC) and an average 9.3% increase in root mean square errors (RMSE), compared with the use of experimental structures. Additionally, AF3-assisted MT-TopLap achieved a PCC of 0.81 when tested with a SARS-CoV-2 HK.3 variant DMS dataset, confirming its capability to accurately predict BFE changes and adapt to new experimental data, thereby showcasing its potential for rapid and effective response to fast viral evolution.

## 1. Introduction

According to the Centers for Disease Control and Prevention (CDC) CDC (2024), the effectiveness of flu vaccines has been much lower than 50% over the past decade, mostly due to the fast evolution of flu viruses and the relatively slow process of vaccine design and manufacture. It takes about 6 months for pharmaceutical makers to produce a flu vaccine, whereas, during this period, the flu virus typically has undergone some crucial mutations at its surface protein. As such, it is extremely valuable to predict the emerging dominant viral variants so that new vaccines can be designed and put into manufacture before a viral variant becomes dominant. During the COVID-19 pandemic, the emerging dominance of Omicron BA.2 Chen and Wei (2022) and BA.4/BA.5 Chen et al. (2022) was accurately forecast nearly 2 months in advance of the World Health Organization (WHO) announcement, offering a life-saving early warning to society. These predictions were based on two natural selection mechanisms of SARS-Cov-2 evolution, namely infectivity strengthening Chen et al. (2020) and antibody resistance (or vaccine breakthrough) Wang et al. (2021). Technically, the accurate forecasting of emerging dominant variants was achieved via the integration of artificial intelligence (AI), topological deep learning (TDL), genotyping of viral genomes extracted from patients, the deep mutational scanning (DMS) of SARS-CoV-2 receptor binding domain (RBD) in complex with human angiotensin-converting enzyme 2 (ACE2), and the three-dimensional (3D) RBD–ACE2 complex structure. Among these, the DMS experiments are typically a bottleneck in response to fast-evolving viruses Starr et al. (2022); Starr et al. (2020); Dadonaite et al. (2024). Indeed, the earliest experimental DMS on the SARS-CoV-2 spike protein RBD mutations was not available until September 2020, 9 months after the outbreak of the COVID-19 pandemic Starr et al. (2020). Additionally, during the global spread of infectious viruses Bergquist et al. (2020), rapid viral evolution poses significant challenges for experimental DMS to keep pace.

Since experiments are time-consuming and high-cost, much effort has been given to the development of effective computational methods for predicting mutational impacts on viral surface protein and human receptor interactions or protein–protein interactions (PPIs) Geng et al. (2019); Pandurangan and Blundell (2020); Geng et al. (2019); Li et al. (2021), Siebenmorgen and Zacharias (2020); Dehouck et al. (2013); Romero-Molina et al. (2022); Xue et al. (2016); Yang et al. (2023); Wang et al. (2020). Remarkably, the TDL approach predicted in-silico DMS of SARS-CoV-2 spike RBD in May 2020 Chen et al. (2020), 4 months earlier than the first experimental DMS Starr et al. (2020). This study identified critical mutation sites at spike protein residue positions 452 and 501, which were predicted to result in significantly more infectious SARS-CoV-2 variants Chen et al. (2020). These sites were subsequently confirmed as key hotspots in several prevailing SARS-CoV-2 variants, including Alpha, Gamma, Delta, Beta, Theta, Mu, Omicron, BA.2, BA.4, BA.5, and all later variants. Nonetheless, experimental DMS is needed for highly accurate and reliable AI-based predictions, such as the aforementioned forecasting of Omicron BA.2 and BA.4/BA.5’s emerging dominance Chen and Wei (2022); Chen et al. (2022).

Introduced in 2017, TDL combines topological data analysis (TDA) with deep learning techniques Cang and Wei (2017). A significant factor in this success is persistent homology Edelsbrunner et al. (2008); Zomorodian and Carlsson (2004), which was used to generate topological fingerprints for predicting binding free energy (BFE) changes upon mutation in PPI complexes Wang et al. (2020). Notably, one of the earliest persistent homology models to integrate with DMS is TopNetmAb, which offered accurate predictions of BFE changes upon mutations in the RBD and ACE2 complexes Chen et al. (2020); Chen et al. (2021).

However, persistent homology has limitations, such as the lack of description of non-topological shape evolution in data. Recently, persistent Laplacian (PL) has been introduced to address these limitations of existing TDA methods Wang et al. (2020). It extracts both topological and geometric information to understand the shape and structure of complex and high-dimensional data. PL is deeply rooted in spectral theory and is part of the family of persistent topological Laplacians, like persistent sheaf Laplacians Wei and Wei (2025) and persistent Mayer Laplacians Shen et al. (2024). The harmonic spectra of PL can fully recover the Betti numbers of persistent homology Edelsbrunner et al. (2008); Zomorodian and Carlsson (2004), a crucial tool in the early days of TDA.

PL has proven effective in scenarios where standard approaches struggle, and it enhances situations where they perform well by providing harmonic and non-harmonic spectral features for TDL models Chen et al. (2022); Wee and Xia (2022); Liu et al. (2022). PL is also used in the latest integration between TDL and pre-trained evolutionary scale modelling (ESM) transformer features Rives et al. (2021) to predict mutation-induced protein solubility changes, establishing a state-of-the-art method for predicting protein–protein BFE changes Wee et al. (2024).

Apart from experimental DMS data, TDL or TopNetmAb also employs the SKEMPI 2.0 database Jankauskaitė et al. (2019), an experimental database for PPI BFE changes upon mutation. SKEMPI 2.0 is used as training data with a multi-task deep learning approach that simultaneously learns single-site mutational patterns with experimental BFE changes and DMS profiles with experimental enrichment ratios. Our recent study on multi-task topological Laplacian (MT-TopLap) Wee et al. (2024), a multi-task deep learning model built by integrating PL, auxiliary, and pre-trained ESM-2 transformer features, revealed a 5% and 21% improvement in root mean square error (RMSE) compared to earlier topology-based models like TopLapNet Chen et al. (2022) and non-topology-based models like mCSM-PPI2 Rodrigues et al. (2019) when predicting BFE changes upon mutation.

Finally, one important factor in ensuring the success of TDL or MT-TopLap for DMS predictions is the availability of high-quality 3D structures of PPI complexes. MT-TopLap requires the 3D structures of new viral variants to achieve accurate predictions. Such structures are typically not available until many months later during pandemics. Without these structures, new experimental DMS data cannot be utilized in TDL models. The rapid mutation rate of SARS-CoV-2 exacerbates this issue, leading to a shortage of both DMS and 3D structural data for emerging SARS-CoV-2 variants. This lack of 3D structures directly poses a significant challenge for TDL and many other machine learning methods, hindering the ability of in-silico DMS to keep pace with viral evolution.

The challenge of lacking 3D structures may be addressed by AlphaFold 3 (AF3), which represents a groundbreaking advancement in the field of computational biology, particularly in the prediction of antibody–antigen or PPIs. Developed by Google DeepMind and Isomorphic Labs Abramson et al. (2024), AF3 builds upon the remarkable success of its predecessors by introducing significant enhancements in protein structure prediction. One of the most notable features of AF3 is its ability to accurately predict the structures of PPI complexes, including those involving DNA, RNA, antibodies, and antigens. Accurate prediction of PPIs is essential for understanding the molecular mechanisms underlying various diseases, developing targeted therapies, and advancing protein engineering efforts.

The capabilities of AF3 in predicting PPIs mark a substantial leap from previous iterations, which primarily focused on predicting the 3D structures of individual proteins. In 2019, AlphaFold’s Jumper et al. (2021) exceptional performance in predicting 58.1% of the test protein structures generated excitement among researchers about the future of AI-driven protein structure prediction. DeepFragLib by Wang et al. Wang et al. (2019) then made a significant advancement in ab initio protein structure prediction. The introduction of AlphaFold marked a transformative shift in how we model protein structures and their interactions Jumper et al. (2021). AlphaFold has since unlocked numerous possibilities in protein folding, protein engineering, and design Wei (2019); Qiu and Wei (2023); Lin et al. (2023); Mirdita et al. (2022), Tunyasuvunakool et al. (2021); Dejnirattisai et al. (2022); Nunes-Alves and Merz (2023). In October 2024, the Nobel Prize in Chemistry was awarded to David Baker for his work on computational protein design, Demis Hassabis, and John Jumper for their revolutionary contributions to AlphaFold.

In this work, we introduce AF3-assisted MT-TopLap to accurately predict the BFE changes upon DMS mutations of SARS-CoV-2 RBD–ACE2 variants. Specifically, we leverage AF3’s predicted structures to extract PL features. As such, we can speed up in-silico DMS predictions by bypassing the experimental determination of PPI complex structures. As a result, the proposed AF3-assisted MT-TopLap can serve the need for a rapid response to fast viral evolution.

We validate our AF3-assisted MT-TopLap approach with four experimental RBD–ACE2 DMS datasets of SARS-CoV-2 and its variants and show that AF3-assisted MT-TopLap maintains strong performance in predicting BFE changes upon DMS mutations, with an average decrease of only 1.1% in Pearson correlation coefficient (PCC) and an average increase of only 9.3% in RMSE, compared to the use of experimental RBD–ACE2 structures. Furthermore, we perform fine-tuning validation of AF3-assisted MT-TopLap using an experimental RBD–ACE2 DMS dataset of SARS-CoV-2 HK.3 Taylor and Starr (2024). AF3-assisted MT-TopLap achieves a PCC of 0.81 and RMSE of 1.10 for this fine-tuning validation. Using AF3’s SARS-CoV-2 RBD–ACE2 complexes extends MT-TopLap’s effectiveness in predicting viral mutation impacts, tracking virus evolution, forecasting emerging dominant variants, and guiding the development of new vaccines.

## 2. Results

In this section, we assess the performance of AF3-assisted MT-TopLap in predicting four SARS-CoV-2 RBD–ACE2 DMS datasets. MT-TopLap integrates PL, auxiliary, and pre-trained transformer features to predict BFE changes upon RBD DMS mutations. Using alternative AF3’s 3D RBD–ACE2 complex structures, we demonstrate that AF3-assisted MT-TopLap can effectively predict the BFE changes upon RBD DMS mutations for SARS-CoV-2 evolution. Specifically, we first collected four experimental RBD–ACE2 DMS datasets, each paired with an experimental 3D SARS-CoV-2 RBD–ACE2 complex structure. Meanwhile, we also create alternative AF3 3D RBD–ACE2 complex structures from the AF3 Server. We evaluate how these alternative AF3 3D RBD–ACE2 complex structures impact our MT-TopLap model’s predictions of BFE changes upon the DMS of RBD, which involves the systematic mutations of each RBD residue into 19 other residues, resulting in thousands of mutations.

The four experimental DMS datasets comprise two datasets from the original SARS-CoV-2 RBD–ACE2 DMSs (6M0J-RBD-1, 6M0J-RBD-2) Starr et al. (2020); Linsky et al. (2020), and datasets from the BA.1-ACE2 complex (7T9L-RBD) and BA.2-ACE2 complex (7XB0-RBD) Starr et al. (2022). Most of the experimental DMS datasets were released during the COVID-19 pandemic to speed up the understanding of how RBD mutations affect SARS-CoV-2 infectivity and antibody resistance. One of the first SARS-CoV-2 experimental datasets used a yeast-surface-display platform to measure the expression of folded RBD protein and its binding to ACE2 Starr et al. (2020). The per-barcode counts from these experiments were used to create a functional score, in the form of log enrichment ratios to estimate the RBD–ACE2 binding affinity values Starr et al. (2020). Consequently, DMS became a crucial tool for examining the SARS-CoV-2 RBD–ACE2 interaction and for designing vaccines and antibodies. Further details can be found in the Supplementary Information.

**Figure 1.**
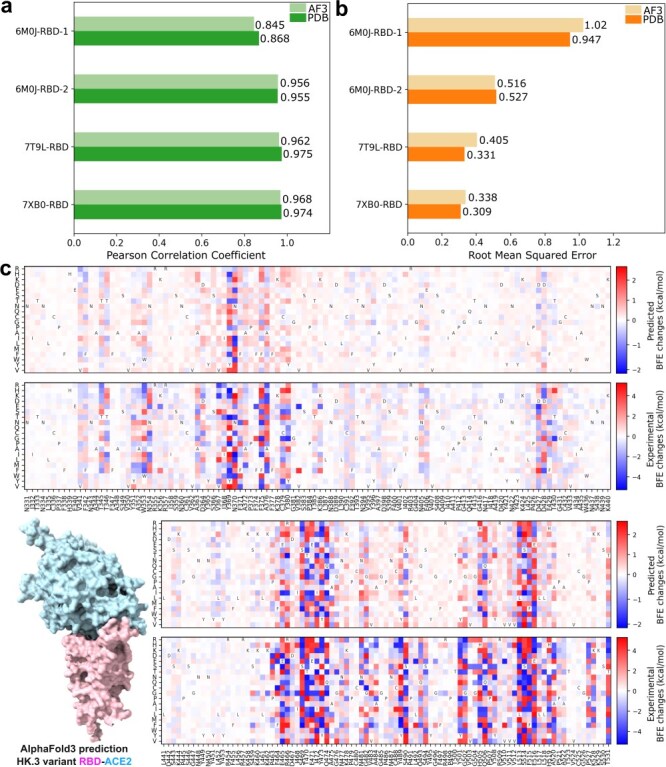
(a) Comparison of AF3-assisted MT-TopLap’s Pearson correlation coefficient (PCC) values for the 10-fold cross-validation on four SARS-CoV-2 RBD–ACE2 DMS datasets with AF3 structures against experimental structures. Higher PCC values indicate better results. (b) Comparison of AF3-assisted MT-TopLap’s root mean square error (RMSE) for the 10-fold cross-validation on four SARS-CoV-2 RBD–ACE2 DMS datasets with AF3 structures against experimental structures. Lower RMSE values indicate better results. (c) The AF3 structural predicted HK.3 RBD–ACE2 complex and its predicted DMS data (top) by AF3-assisted MT-TopLap. DMS predictions are obtained by performing a 10-fold cross-validation using the experimental DMS data (bottom). The *x*-axis labels represent the RBD residues and its wild amino acid types. The *y*-axis labels represent the mutant amino acid types.

Figures 1(a) and (b) show the comparative analysis of the performance of AF3-assisted MT-TopLap for the four SARS-CoV-2 RBD–ACE2 DMS datasets. AF3-assisted MT-TopLap only displayed an average decrease of 1.1% in PCC when compared to using Protein Data Bank (PDB) structures. Nonetheless, the four SARS-CoV-2 RBD–ACE2 datasets displayed an average of 9.3% increase in RMSE when validated with AF3-assisted MT-TopLap. AF3-assisted MT-TopLap also achieved the best performance in the validation test for 7XB0-RBD with a PCC of 0.968, only 0.6% lower than the performance using PDB complexes. AF3-assisted MT-TopLap’s predicted BFE changes for 7XB0-RBD obtained the lowest RMSE of 0.338 kcal/mol as compared to the other three DMS datasets. This indicates that the AF3-assisted MT-TopLap is still highly effective in predicting BFE changes caused by BA.2 RBD DMS mutations.

To enhance AF3-assisted MT-TopLap’s capabilities using experimental DMS data, we use transfer learning to update AF3-assisted MT-TopLap by training it with the latest experimental DMS data. This process is particularly important for DMS in SARS-CoV-2 pandemic research, as experimental DMS approaches can be slow and inefficient for tracking the rapid viral evolution. Recently, transfer learning has been applied to fine-tune MT-TopLap to predict DMS in SARS-CoV-2 S protein RBD binding to ACE2 in various animal species like bats, cats, deer, and hamsters due to RBD mutations Wee et al. (2024). This enabled MT-TopLap to identify potential RBD mutations that enhance human–animal cross-transmission Wee et al. (2024). With AF3, we can finetune MT-TopLap with new experimental DMS data even though high-quality 3D SARS-CoV-2 RBD–ACE2 variants are unavailable in PDB. To finetune AF3-assisted MT-TopLap, we collected a recent experimental DMS dataset based on the SARS-CoV-2 HK.3 variant RBD–ACE2 complex Taylor and Starr (2024), which does not have an available high-quality 3D HK.3 RBD-human ACE2 complex in PDB (accessed PDB on 5th Oct 2024). HK.3 is one of the XBB subvariants and is known for its ‘FLip’ substitutions with mutations S:L455F and S:F456L. Similarly, we use the AlphaFold Server to predict the 3D structure of HK.3 variant RBD–ACE2 complex. Figure 1(c) shows the RBD–ACE2 complex predicted by AF3, the comparison between experimental BFE changes (converted from enrichment ratios) (Figure 1(c) bottom) and predicted BFE changes induced by HK.3 RBD mutations (Figure 1(c) top). The predicted BFE changes are obtained by performing a 10-fold cross-validation after MT-TopLap has been pre-trained with the SARS-CoV-2 RBD–ACE2 datasets and the S8338 dataset. The patterns of the predicted DMS are observed to resemble the patterns of the converted BFE changes with a PCC of 0.81 and an RMSE of 1.10. On the whole, MT-TopLap’s prediction still reflects the overall trend and this supports the use of latest experimental DMS data and AF3 structures to update the MT-TopLap model.

## 3. Discussion

### 3.1 Analysis of BFE changes by structural regions

**Figure 2.**
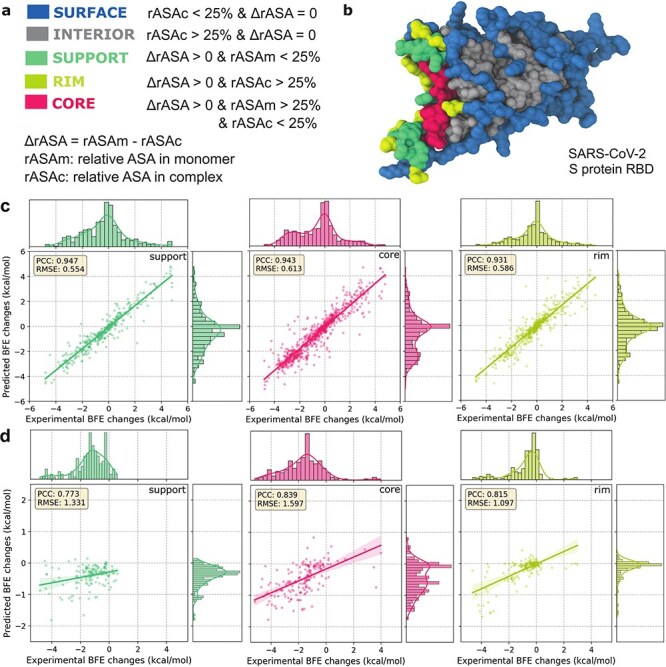
(a) Definitions of structural regions based on relative ASA in monomer and complex Levy (2010). (b) Structural regions on the Spike protein RBD (PDBID: 6M0J Lan et al. (2020)). Amino acids are assigned to the surface, interior, support, rim, and core based on the rASA in monomer and complex. Structures are plotted by VMD Humphrey et al. (1996). (c) The 10-fold cross-validations for the four AF3-assisted SARS-CoV-2 RBD–ACE2 datasets displayed an average PCC of 0.933 and an average RMSE of 0.570 (see Figure 1(a)-(b)). The combined prediction results for different residue region types for four AF3-assisted SARS-CoV-2 RBD–ACE2 datasets according to Fig. 1(a) with PCCs of 0.947, 0.943, and 0.931 for the binding interfaces: support, core, and rim, respectively. The average enrichment of the experimental DMS data is compared. (d) The 10-fold cross-validations of DMS on the HK.3 RBD–ACE2 Taylor and Starr (2024) show a PCC of 0.81 and an RMSE of 1.10. Prediction results for different residue region types according to Fig. 1(a) with PCCs of 0.773, 0.839, and 0.815 for the binding interfaces: support, core, and rim, respectively. The average enrichment of the experimental DMS data is compared.

In this section, we analyse the results by categorizing the mutation residues based on their structural regions. The relative accessible surface area (rASA) values are calculated based on the definitions in Figure 2(a) and this categorizes each mutation residue into interior and surface structural regions depending on the rASA values monomeric and PPI states Levy (2010). Mutated residues within the binding interface significantly affect the BFE changes of the PPI and are further categorized into support, core, and rim regions Bogan and Thorn (1998). Figure 2(b) illustrates the structural regions for the original SARS-CoV-2 S protein RBD. This approach has been strongly validated in previous research as effective for identifying protein interaction interfaces. The variability in rASA calculations enables the dynamic classification of mutation residues in PPIs, even underscoring the viral adaptability in SARS-CoV-2 RBD–ACE2 interactions.

Predicting BFE changes induced by DMS mutations within the binding interface is most vital in assessing AF3-assisted MT-TopLap’s performance. In this study, experimental log enrichment ratios were converted into binding free energies, albeit with errors. Some discrepancies were noted in mutations occurring both inside and on the surface. Figure 2(c)-(d) illustrates the 10-fold cross-validation performance of AF3-assisted MT-TopLap in the support, core, and rim regions of the binding interface. In Figures 2(c) and (d), both the four SARS-CoV-2 RBD–ACE2 data and the HK.3 RBD–ACE2 DMS data displayed high correlations in the support, rim, and core regions. This indicates that AF3-assisted MT-TopLap performs well in predicting BFE changes upon mutations in the binding interface of PPIs. The high correlations for the HK.3 RBD–ACE2 DMS data also suggest that finetuning MT-TopLap remains robust in predicting the binding interface of HK.3 RBD–ACE2 complex.

Next, we examine the results of AF3-assisted MT-TopLap for mutated residues beyond the binding interface. Figure S2 highlights the average PCC of AF3-assisted MT-TopLap for predicted BFE changes due to surface and interior mutations. Across the four DMS datasets, high correlations were observed for surface and interior mutations (see Figure S2). However, the HK.3 RBD–ACE2 DMS dataset showed weaker PCCs, likely due to significant negative BFE changes (ranging from -5 to 0) for surface and interior mutations, while predicted values ranged from -2 to 0. Thus, the PCCs for surface and interior regions were down to 0.557 and 0.837, respectively. This finding aligns with earlier studies where the TDL-DMS model also reported weaker correlations for surface and interior mutations Chen et al. (2023). Nonetheless, AF3-assisted MT-TopLap performed well on the binding interface of the protein–protein complex, which is most important for understanding mutational impacts on PPI systems (see Figure 2).

### 3.2 Analysis of BFE changes by mutation types

The prediction results are also analysed by categorizing over different mutation types. The pattern of predicted BFE changes based on various mutation types is a crucial element in protein design, particularly in the development of monoclonal antibodies (mAbs). Here, we assess how well our predictions resemble the distribution in experimental data by examining the behaviour of our model for 20 distinct amino acid types across the four SARS-CoV-2 RBD–ACE2 DMS datasets.

**Figure 3.**
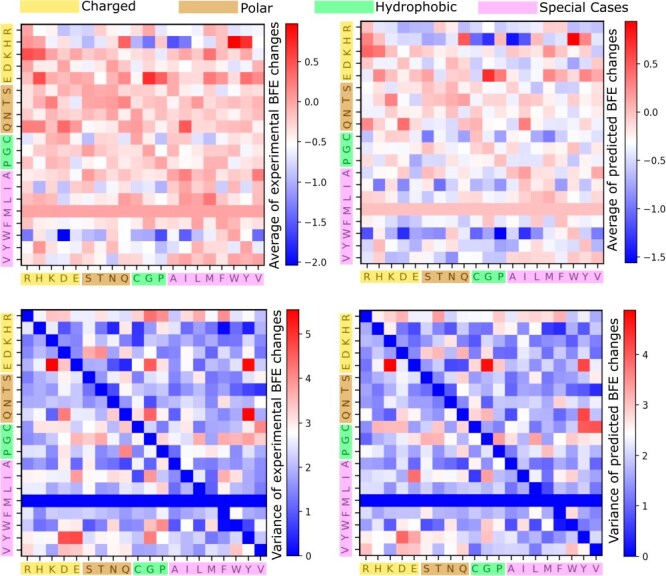
A comparison of average experimental and predicted BFE changes following mutations associated with different amino acid types for all the four SARS-CoV-2 RBD–ACE2 DMS datasets. The *x*-axis labels the residue type of the original RBD amino acids, whereas the *y*-axis labels the residue type of the mutant. Note that there is no amino acid MET (M) on RBD mutations. Top: Average BFE changes following mutation. Bottom: Variance of BFE changes following mutation. Left: Experimental values. Right: Predicted values.

Using AF3 structures, the prediction patterns remain closely aligned with the experimental data, both in terms of average BFE changes upon mutation and their variance (see Figure 3). The average of predicted BFE changes upon mutation exhibits primarily a negative change, as evidenced by the predominance of negative range in the colour bars. Additionally, the variance of the predicted BFE changes is generally lower than the variance of the experimental values, as indicated by the shift in the colour bar range. This implies that while achieving highly accurate BFE changes upon mutation is crucial, maintaining a level of diversity comparable to the experimental data remains a challenging endeavour.

In terms of amino acid sizes, we categorize the 20 amino acids into charged, polar, hydrophobic, and special-case groups. In Figure 3, we observe that mutations from charged or polar residues to other types generally lead to the most positive BFE changes. This indicates increased stability within the PPI systems, such as when mutating from K, E, and T to other amino acid types. On the other hand, higher instability is predicted for mutations from W to other residues as shown by the deep blue squares. This analysis provides insights into the molecular dynamics and potential mutation effects on SARS-CoV-2 RBD–ACE2-binding interface.

### 3.3 Tracking SARS-CoV-2 variants

SARS-Cov-2 evolution is driven by two natural selection mechanisms, namely infectivity strengthening Chen et al. (2020) and antibody resistance (or vaccine breakthrough) Wang et al. (2021). These mechanisms were used to predict emerging dominant SARS-CoV-2 variants Chen and Wei (2022); Chen et al. (2022).

**Figure 4.**
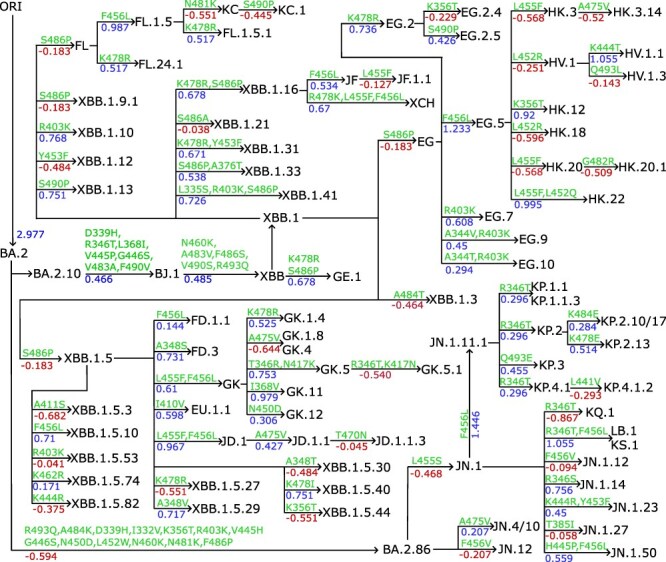
The lineage of Omicron XBB and BA.2.86 subvariants. RBD mutation-induced BFE changes are predicted with MT-TopLap after finetuning with HK.3 variant DMS dataset. RBD mutation-induced BFE changes (kcal/mol) are marked from parent generations to children as well as mutations. ORI represents the original SARS-CoV-2 virus.

In the results, we finetuned AF3-assisted MT-TopLap with the HK.3 variant DMS data. This expanded AF3-assisted MT-TopLap’s training data but also enhanced its capabilities in tracking the recent evolutionary trajectories of SARS-CoV-2 virus. Figure 4 shows the annotation tree plots of BA.2.10 and BA.2.86 subvariants. RBD mutations from parent generations to their children are marked on the arrows and predicted BFE changes (kcal/mol) induced by the corresponding RBD mutations. BFE changes from parent generations to their children are summed up by the BFE changes upon each RBD mutation. This means that the BFE changes from parent generations to their children which undergoes multiple mutations is calculated by summing up the BFE changes upon each RBD mutation. Positive BFE changes upon mutation are coloured blue while negative values are coloured red.

The BA.2.10 brought about the XBB wave, a series of SARS-CoV-2 variants that emerged from a recombination event between two Omicron subvariants, BA.2.10.1 and BA.2.75. The XBB lineage was first reported in India during the summer of 2022. One of the most notable variants within this lineage is XBB.1.5, also known as the ‘Kraken’ subvariant. It was first detected in the United States in October 2022. XBB.1.5 is highly transmissible and has shown significant immune evasion capabilities, making it a concern for global health authorities. As a result of RBD mutations, XBB.1.5 generated 24 subvariants (see Figure 4). Thereafter, the subvariant EG.5, a descendant of XBB.1.9.2, was detected in early February 2023. EG.5 became a pre-dominant subvariant in August 2023 Taylor and Starr (2024). Notably, HK.3 is a descendant of EG.5.1 and is more transmissible due to the mutations S:L455F and S:F456L.

Apart from the competing strains of BA.2.10, i.e. EG.5 and HK.3, a descendant of BA.2 known as BA.2.86, was detected in August 2023 and exhibits as many as 30 mutations. In Figure 4, BA.2 requires 13 RBD mutations to become BA.2.86. It was only until early 2024 that a BA.2.86 subvariant, JN.1, became a widely circulating variant in the United States. As of 28th June 2024, both BA.2.86 and JN.1 are variants of interests (VOIs) under the WHO. As of 24th September 2024, JN.1 subvariants such as KP.2, KP.3 and LB.1 are circulating variants monitored by the WHO.

Interestingly, the Q493E mutation has been observed to decrease BFE changes in all previous SARS-CoV-2 variants Taylor and Starr (2024). However, in the current KP.3 variant, when Q493E is combined with L455S and F456L mutations, the effect has reversed thus promoting an increase in BFE changes Taylor and Starr (2024). This is consistent with Figure 4 where a positive BFE change of 0.455 kcal/mol due to Q493E mutation led to the KP.3 variant. However, Figure 4 also shows that there are other subvariants of KP.2 (e.g. KP.2.10/17 and KP.2.13) which generated a higher BFE change but are currently not monitored by the WHO. Likewise, LB.1 has a lower BFE change as compared to JN.1.11.1, and yet it is a circulating variant monitored by the WHO. A recent study indicated that an epistatic drift exists in the RBD mutational effects of the current SARS-CoV-2 virus evolution Taylor and Starr (2024). This suggests that the mutational effects on KP.2, KP.3, and LB.1 are possibly influenced by new the antibody resistance mechanism Wang et al. (2021), which is beyond the scope of the present work.

Although AF3 could mitigate the shortage of experimental 3D structures needed for robust deep learning frameworks, one potential limitation still lies in the quality of AF3’s SARS-CoV-2 RBD–ACE2 complexes. Table S2 shows the structural alignment RMSD, interface predicted template modelling (ipTM) and predicted template modelling (pTM) scores of the AF3’s SARS-CoV-2 RBD–ACE2 complexes. RMSD scores are obtained by aligning AF3 complexes with the original PDB structures. Both ipTM and pTM are confidence metrics used to evaluate the AF3 prediction accuracy. Note that lower RMSD, high pTM and high ipTM indicates better performance. In Table S2, all AF3 structures have an RMSD < 1Å when structurally aligned with respect to the RBD. The high pTM scores indicate that the overall predicted fold for the complex might be similar to the true structure but the low ipTM scores indicate the inaccuracies in the overall RBD–ACE2 complex prediction. Here, a poor ipTM score does not necessarily translate into poor RMSD performance. One possible explanation could be the limitations AF3 possess when it comes to predicting non-canonical amino acids. In SARS-CoV-2 RBD–ACE2 complexes, this indicates that AF3 cannot predict the hACE2 N90-linked glycan residues in the complex. A recent study discovered that the specific binding of the BA.2 residue T415 to the hACE2 N90-glycan, along with the increased affinity of the BA.2 RBD for hACE2, suggesting that the N90-glycan/T415 interaction promotes the binding of the BA.2 RBD to hACE2 Li et al. (2022). This suggest that the AF3’s SARS-CoV-2 RBD–ACE2 complexes still needs room for improvement in its overall complex prediction.

Nevertheless, leveraging AF3’s SARS-CoV-2 RBD–ACE2 complexes potentially enhances MT-TopLap’s ability to predict the impacts of viral mutations, monitor virus evolution, anticipate emerging dominant variants, and inform the development of new vaccines. In AF3-assisted MT-TopLap model, the combination of PL features with auxiliary, and pre-trained transformer features helps to offset the structural inaccuracies due to AF3. However, ongoing advancements in experimental structure determination methods, like the significant improvements in cryo-electron microscopy and tomography, are still necessary to supply abundant new 3D structures to enhance the generalization capability of AF3-assisted MT-TopLap.

## 4. Methods

Apart from the four experimental SARS-CoV-2 RBD–ACE2 DMS datasets used to validate AF3-assisted MT-TopLap, the largest mutation-induced BFE changes database, SKEMPI 2.0 Jankauskaitė et al. (2019) is also used as multi-task training data.

As mentioned in the results section, AlphaFold Server was used to predict the 3D protein–protein complexes for the four experimental SARS-CoV-2 RBD–ACE2 DMS data. Experimental SARS-CoV-2 RBD–ACE2 DMS datasets used are SARS-CoV-2 DMS obtained from RBD-induced mutations for original SARS-CoV-2 RBD binding to ACE2 Starr et al. (2020); Linsky et al. (2020). Additionally, mutational scanning of BA.1 and BA.2 RBD binding to ACE2 is also included Starr et al. (2022). Comprehensive details about the experimental SARS-CoV-2 RBD–ACE2 DMS datasets and the SKEMPI 2.0 database are available in the Supporting Information.

### 4.1 AF3-assisted MT-TopLap for predicting BFE changes upon mutation

The MT-TopLap model was built by integrating PL, auxiliary, and pre-trained transformer features to predict PPI BFE changes upon mutation Wee et al. (2024). In this work, an AF3-assisted MT-TopLap is developed to predict BFE changes upon mutation with AF3-generated PPI complex structures. As shown in Figure S1, the AF3-generated 3D SARS-CoV-2 RBD–ACE2 complex is used to generate PL features and auxiliary features. PL features provide a topological representation, using element- and site-specific atom sets to simplify the structural complexity of protein–protein complexes and encode vital biological information into topological invariants. Previously, a major success for applying PL features is the ability to use topological deep learning model to accurately predict the dominance of Omicron BA.4 and BA.5 nearly 2 months before WHO made the official announcement Chen et al. (2022). The ESM-2 pre-trained transformer is used to convert amino acid sequence of the PPI into a pre-trained transformer embedding. All these features are concatenated and fed into MT-TopLap to predict the BFE change upon mutation.

The deep neural network model in MT-TopLap’s architecture consists of six hidden layers with 15,000 neurons in each layer and generates an output channel for each dataset. For the validation process, we perform dataset-level 10-fold cross-validation on four experimental SARS-CoV-2 RBD–ACE2 datasets. The learning rate is set to 0.0001 and 500 epochs are used for the pre-training while 200 epochs are used for the validation step. The output for each dataset is represented by an individual output channel for training the experimental enrichment ratios (see Figure S1). For the fine-tuning process, the weights and biases for the second and third hidden layers are frozen. Here, the learning rate is set to 0.0001 and 200 epochs are used for fine-tuning. Details of the method can be found in the literature Wee et al. (2024). Specific details for 10-fold cross-validations and the finetuning performed in this work are given in the Supporting Information.

### 4.2 Persistent Laplacian features

One of the main descriptors used in AF3-assisted MT-TopLap is the PL feature vectors. We now outline the key mathematical principles behind the PL descriptors in AF3-assisted MT-TopLap. This involves describing the simplicial complex and PL methods, emphasizing their importance in capturing harmonic and non-harmonic spectral properties crucial for characterizing SARS-CoV-2 RBD–ACE2 interactions. Details about the auxiliary, persistent homology and the ESM-2 transformer features can be found in the Supporting Information.

To construct PL features for the RBD–ACE2 complex, we categorize the atoms in the complex into specific site-based subsets. These include mutation site atoms $\mathcal{A}_m$, atoms within a distance *r* of mutation site $\mathcal{A}_{mn}(r)$, atoms from the RBD binding site $\mathcal{A}_{\text{RBD}}(r)$, and atoms from the ACE2 binding site $\mathcal{A}_{\text{ACE2}}(r)$. We also classify atoms into different element-specific subsets, such as $\{\text{C, N, O}\}, \mathcal{A}_{\text{ele}}$. These partitions are key for PPI model characterization, as different atom combinations capture various interaction types. For instance, subsets $\mathcal{A}_{\text{C}}\cap \mathcal{A}_{\text{RBD}}(r)$ and $\mathcal{A}_{\text{C}}\cap \mathcal{A}_{\text{ACE2}}(r)$ form hydrophobic $\rm C- \rm C$ RBD–ACE2 interactions, while $\mathcal{A}_{\text{N}}\cap \mathcal{A}_{\text{RBD}}(r)$ and $\mathcal{A}_{\text{O}}\cap \mathcal{A}_{\text{ACE2}}(r)$ result in hydrophilic $\rm N- \rm O$ RBD–ACE2 interactions.

In order to characterize the RBD–ACE2 binding interactions, we also modify the standard Euclidean distance matrix DE such that it excludes interactions between both atoms found in the RBD or both in the ACE2. Specifically, for interactions between atoms $A_i$ and $A_j$ in sets $\mathcal{A}$ and $\mathcal{B}$, DI is defined as follows:


(1) $$\mathrm{DI}(A_i,\;A_j)\;=\left\{\begin{array}{l}\infty,\;\text{if}A_i,A_j\in\mathcal A\text{or if}A_i,A_j\in\mathcal B\\\mathrm{DE}(A_i,\;A_j),\;\mathrm{otherwise}\end{array}\right.$$

where $\rm DE(.,.)$ is the Euclidean distance between the two atoms.

For each site and element-specific subset, the 3D atom positions generate point clouds which are then used to build simplicial complexes. A set of $k$ + 1 atoms from a site/element-specific subset forms $k+1$ independent points, denoted as $S=\{v_0, v_1, v_2, \cdots, v_k\}$. The convex hull of $k+1$ affinely independent points forms a *$k$*-simplex: with a point being a 0-simplex, an edge being a 1-simplex, a triangle being a 2-simplex, and a tetrahedron being a 3-simplex, while higher dimensions form $k$-simplices. A simplicial complex is created from the aggregation of these finite simplices Munkres (2018); Zomorodian (2005); Edelsbrunner and Harer (2010); Mischaikow and Nanda (2013). There are various methods for constructing simplicial complexes. For generating our PL-based features, we utilized the Vietoris-Rips complex for dimension 0 and the Alpha complex for dimensions 1 and 2. The Vietoris-Rips (VR) complex forms simplices by connecting subsets of points with diameters not exceeding a given threshold. On the other hand, the Alpha complex is derived from Delaunay triangulation, constrained by a radius not exceeding a specified threshold, which subdivides the convex hull of a point set into triangles.

For a simplicial complex *$K$*, a *$k$*-th chain *$c_k$* is the formal sum of *$k$*-simplicies in $K$, i.e. $c_k = \sum_i \alpha_i\sigma_i^k$. A boundary operator $\partial_k: C_k \rightarrow C_{k-1}$ defined on a *$k$*-th chain $c_k$ is


$$ \partial_k c_k = \sum_{i=0}^k \alpha_i\partial_{k}\sigma_i^k, $$

such that the boundary of a boundary is empty, i.e. $\partial_{k-1}\partial_k = \varnothing $. By defining the adjoint of $\partial_k$, i.e. $\partial_{k}^*:C_{k-1}\to C_{k}$, we have $\partial_{k}^*$ satisfying the inner product relation $\langle \partial_k(f), g \rangle=\langle f, \partial_{k}^*(g) \rangle,$ for every $\textstyle f\in C_k,g\in C_{k-1}$. Then the $k$-combinatorial Laplacian or the topological Laplacian is a linear operator $\Delta_k: C_k(K) \rightarrow C_k(K)$


(2) $$ \Delta_k := \partial_{k+1}\partial_{k+1}^* + \partial_{k}^*\partial_{k}. $$

In terms of matrix representations, we define $\mathbf{B}_k$ to be an $m\times n$ matrix representation of the boundary operator under the standard bases $\{\sigma_i^k\}^n_{i=1}$ and $\{\sigma_j^{k-1}\}^m_{j=1}$ of $C_k$ and $C_{k-1}$. Similarly, the matrix representation of $\partial_{k}^*$ is the transpose matrix $\mathbf{B}_k^\top$, with respect to the same ordered bases of the boundary operator $\partial_k$. Hence, the $k$-combinatorial Laplacian exhibits an $n\times n$ matrix representation $\mathbf{L}_k$ and is given by


(3) $$ \mathbf{L}_k = \mathbf{B}_{k+1}\mathbf{B}_{k+1}^\top + \mathbf{B}_k^\top \mathbf{B}_k. $$

In the case $k=0$, then $\mathbf{L}_0 = \mathbf{B}_1\mathbf{B}_1^\top$ since $\partial_{0}$ is a zero map.

In our model, the eigenvalues of combinatorial Laplacian matrices are key topological features, independent of orientation choice Horak and Jost (2013). The multiplicity of zero eigenvalues of $\mathbf{L}_k$ corresponds to the $k$-th Betti number $\beta_k$, which describes the $k$-dimensional holes in a simplicial complex Eckmann (1944). Specifically, $\beta_0,$  $\beta_1$ and $\beta_2$ represent the number of independent components, loops, and voids, respectively. These Betti numbers provide critical insights into the fundamental structure of the protein–protein interaction, identifying loops and voids within the PPI system.

Zero eigenvalues in the Laplacian matrix signify the harmonic spectra, representing stable features like connected components and cycles that persist across different scales. Non-zero eigenvalues represent the non-harmonic spectra, revealing more transient and intricate details of the molecular shape and interactions that are not captured by Betti numbers alone. These non-harmonic spectra offer additional homotopic shape information, crucial for a comprehensive understanding of the biomolecular interaction dynamics.

One simplicial complex is insufficient to capture all topological information from a protein–protein interaction structure. By integrating combinatorial Laplacian and multiscale filtration, we track changes in harmonic and non-harmonic spectra by varying a filtration parameter such as radii/diameter for the VR complex. For an oriented simplicial complex $K$, filtration generates a nested sequence of simplicial complexes $(K_t)^m_{t=0}$:


$$ \varnothing = K_0 \subseteq K_1 \subseteq \cdots \subseteq K_m=K. $$

PL produces a sequence of simplicial complexes as the filtration parameter increases, allowing us to generate a sequence of combinatorial Laplacian matrices $\mathbf L_k^0,{\mathbf L}_k^1,{\mathbf L}_k^2,\mathbf L_k^3,\cdots,\mathbf L_k^n$, where $\mathbf{L}_{k}^t=\mathbf{L}_k(K_t)$. By altering the filtration parameter and performing diagonalization on the $k$-combinatorial Laplacian matrix, we can examine the topology and spectrum characteristics. The eigenvalues of $\mathbf{L}_k(K_t)$ can be arranged in ascending order:


$$ \text{Spectra}(\mathbf{L}_k^t) = \{(\lambda_1)_k^t, (\lambda_2)_k^t, \cdots, (\lambda_n)_k^t\}, $$

where $\mathbf{L}_k^t$ is an $n\times n$ matrix. Additionally, the $p$-persistent $k$-combinatorial Laplacian can be extended based on the boundary operator.

When generating features, we take both harmonic and non-harmonic spectra into account for each PL in zero dimensions. Using filtration with the Rips complex and DI distance, we generate 0-dimensional PL features ranging from 0Å to 6Å with a grid size of 0.5Å. For non-harmonic spectra, we count the occurrences and compute seven statistical values: sum, minimum, maximum, mean, standard deviation, variance, and the sum of squared eigenvalues. This results in eight statistical values for each of the nine atomic pairs, producing a total of 72 features for a protein in zero dimensions. When concatenated for different dimensions of the wild type and mutant, the total 0-dimensional PL-based feature size is 1872.

For one- and two-dimensional PL features, we use the Alpha complex with DE distance for filtration. Due to the limited number of atoms in local protein structures that can form only a few high-dimensional simplexes, we focus on the harmonic spectra of PLs. This captures topological invariants of high-dimensional interactions, providing a comprehensive view of the molecular structure. Using GUDHI Maria et al. (2014), the persistence of harmonic spectra is represented by persistent barcodes. We generate topological feature vectors by computing statistics of bar lengths, births, and deaths, excluding bars shorter than 0.1Å as they lack clear physical significance. The statistics computed include the sum, maximum, and mean of bar lengths; minimum and maximum of bar birth values; and minimum and maximum of bar death values. Each set of point clouds results in a seven-dimensional vector. These features are calculated for nine single atomic pairs and one heavy atom pair, yielding 140 features for one- and two-dimensional PL vectors of a protein. When combined for different dimensions of wild type, mutant, and their differences, the total higher-dimensional PL-based feature size is 420.

The PL features generated in this manner provide a rich and detailed representation of the RBD–ACE2 interactions, capturing both local and global structural features that are critical for accurate modelling. These features enable us to perform critical analyses based on the predicted BFE changes caused by RBD mutations, thereby leveraging the deep contextual information encoded in the RBD–ACE2 binding domain. By integrating harmonic and non-harmonic spectra and considering different dimensions, we obtain a comprehensive understanding of the topological and geometric characteristics of the SARS-CoV-2 RBD–ACE2 system, facilitating accurate predictions and insights into its behaviour and interactions. Further details about PL methods and their applications are reported in Wang et al. (2020).

## 5. Conclusion

Our ability to rapidly respond to viral evolution underpins the health and well-being of the human race. A topological deep learning (TDL)-based approach has a proven track record in forecasting emerging dominant SARS-CoV-2 variants, such as Omicron BA.2 Chen and Wei (2022) and BA.4/BA.5 Chen et al. (2022), approximately 2 months in advance. However, such forecasting is often bottlenecked by the lack of experimental DMS data and the three-dimensional (3D) structures of protein–protein interaction (PPI) complexes for fast-evolving viruses. We address this challenge by leveraging AF3 to predict the PPI complexes. Specifically, we propose an AF3-assisted MT-TopLap model to predict DMS data and forecast emerging viral variants. AF3-assisted MT-TopLap leverages TDL and PLs to capture both topological and geometric features of PPI complexes. It accurately predicts both the DMS profiles and the BFE changes of the PPI complexes for viral variants. We validate the proposed AF3-assisted MT-TopLap strategy by using four experimental DMS datasets on the SARS-CoV-2 spike RBD bound to the human angiotensin-converting enzyme 2 (ACE2). With 3D PPI complexes generated by AF3, AF3-assisted MT-TopLap achieves robust predictive performance, showing minimal deviation from results obtained using high-quality experimental 3D structures. Our model’s success extends to new variants, including the SARS-CoV-2 HK.3 variant, where it achieved a PCC of 0.81. This highlights AF3-assisted MT-TopLap’s ability to adapt to emerging viral data and respond effectively to fast viral evolution.

## Supplementary Material

veaf026_Supp

## Data Availability

The training data used in this work consists of the comprehensive four SARS-CoV-2 RBD–ACE2 datasets which are readily available in https://github.com/ExpectozJJ/MT-TopLap/tree/main/AF3_SARS-CoV-2. The AF3 SARS-CoV-2 RBD-ACE2 complexes can also be downloaded from https://github.com/ExpectozJJ/MT-TopLap/tree/main/AF3_SARS-CoV-2. The original PDB files used in this work can be downloaded from the official Protein Databank: https://www.rcsb.org/. The SKEMPI 2.0 database is also readily available from https://life.bsc.es/pid/skempi2.

## Code availability.

The codes for 10-fold cross-validation and finetuning tasks can be obtained from the following source: https://github.com/ExpectozJJ/MT-TopLap/tree/main/AF3_SARS-CoV-2. Details about the feature generation and MT-TopLap’s model architecture is available in Supporting Information.
