# Supplementary material for: Rapid response to fast viral evolution using AlphaFold 3-assisted topological deep learning
Source: Virus Evol. 2025 Apr 29;11(1):veaf026. doi: 10.1093/ve/veaf026 (PMC12063592; doi:10.1093/ve/veaf026)
Supplement: veaf026_Supp [file veaf026_supp.zip › suppl_data/DMS_AF3_SI_v8.pdf]

Supporting Information for
Rapid response to fast viral evolution using AlphaFold 3-assisted topological deep learning

JunJie Wee[1], and Guo-Wei Wei[1,2,3,*]
[1]Department of Mathematics, Michigan State University, East Lansing, MI 48824, USA
[2]Department of Biochemistry and Molecular Biology,
Michigan State University, East Lansing, MI 48824, USA
[3]Department of Electrical and Computer Engineering,
Michigan State University, East Lansing, MI 48824, USA
[*] Address correspondences to Guo-Wei Wei. E-mail: weig@msu.edu

Contents

1 MT-TopLap Framework **2**
1.1 Multitask Deep Learning Algorithm 4
1.2 Assessment of AF3-assisted MT-TopLap's performance beyond binding interface 5

2 Feature Generation Process **5**
2.1 ESM-2 Transformer Features 5
2.2 Auxiliary descriptors 7
2.3 Harmonic Spectral Features 10
2.4 Software and resources 10

1 MT-TopLap Framework

In this study, four SARS-CoV-2 RBD-ACE2 datasets were used to validate the performance of AF3-assisted MT-TopLap, as outlined in Table S1. Recently, MT-TopLap has been used to predict RBD mutations that could enhance SARS-CoV-2 cross-species transmission[1]. Additionally, some of these datasets have been employed in the TDL-DMS model to predict deep mutational scanning datasets[2]. One of the earliest datasets, 6M0J-RBD-1, was released early in the COVID-19 pandemic. This dataset contains experimental DMS results for the original RBD-ACE2 complex, conducted by T. Starr et. al.[3]. The experiments used a yeast-surface-display platform to assess the expression of folded RBD protein and its interaction with ACE2. By counting each barcode obtained during the experiments, a functional scoring method was applied to determine RBD-ACE2 binding affinity. Chen et al. conducted an *in silico* DMS prediction using the TDL-DMS model to guide future experiments and analyses by understanding the effects of mutations on SARS-CoV-2 infectivity and antibody resistance[2].

Dataset	No. of samples	PDB ID
6M0J-RBD-1 [3]	3669	6M0J [5]
6M0J-RBD-2 [4]	1539	6M0J [5]
7T9L-RBD [7]	3800	7T9L [8]
7XB0-RBD [7]	3686	7XB0 [9]

Table S1: The size of each SARS-CoV-2 RBD-ACE2 dataset used in MT-TopLap for validation.

Besides the DMS training data, AF3-assisted MT-TopLap is also trained with the S8338 dataset. Derived from the SKEMPI 2.0 database[10], S8338 is the largest PPI dataset with mutation-induced binding free energy (BFE) changes, containing 8338 entries of single-point mutations, including reverse mutations. SKEMPI 2.0 improves the original SKEMPI database by incorporating new mutations from AB-bind, PROXiMATE, and dbMPIKT. Models like mCSM-PPI2 have been validated using selected single-point mutations from SKEMPI 2.0. Overall, the S8338 dataset comprises 8338 samples across 319 PPI complexes. For reverse mutations, the BFE changes are assigned as the negative equivalents of the original BFE changes.

Figure S1 illustrates how AF3-assisted MT-TopLap predicts BFE changes upon mutation from new experimental DMS data that does not have structures available in the PDB. For each PPI complex, the amino acid sequences are fed into AlphaFold3 to predict its 3D complex structure. To assess the accuracy of AlphaFold3's predicted structures, we conduct a structural alignment between AF3 structures and the original ones in PDB. We used the matchmaker program in ChimeraX[11] to perform this structural alignment. Table S2 shows the alignment RMSDs for each of the PDB IDs predicted. The RMSDs were all found to be less than 1Å, which indicates a relatively accurate structural prediction by AF3. On a separate note, AF3's complexes has previously been found to be unreliable for some intrinsically disordered regions[12].

Thereafter, Persistent Laplacian features, auxiliary features and ESM-2 transformer features are generated and concatenated to form a long feature vector for each mutation sample. Here, Persistent Laplacian plays a crucial role in MT-TopLap's successful DMS data predictions by effectively characterizing proteins. One of the major successes of Persistent Laplacian features is that it has previously predicted Omicron BA.4 and BA.5 as dominant variants two months before WHO's June 2022 confirmation [13].

Table S2: The structural alignment RMSDs (Å) between the AF3's predictions and the original structures in PDB. The pTM and ipTM scores of AF3's predictions are also reported.

PDB ID	RMSD (Å)	pTM	ipTM
6M0J	0.524	0.7	0.13
7T9L	0.584	0.58	0.36
7XB0	0.573	0.73	0.13

The combined feature vector is then fed into MT-TopLap to train the experimental enrichment ratios of the DMS data. MT-TopLap outputs the converted BFE changes upon mutation.

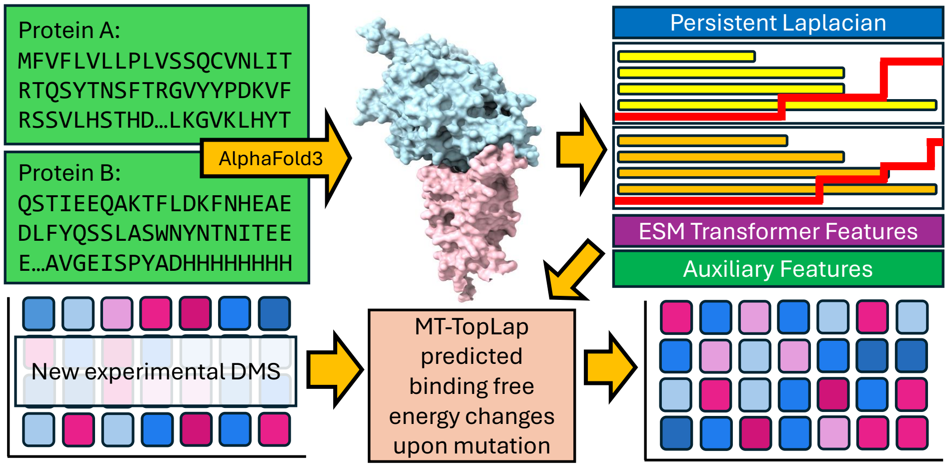

Figure S1: An illustration of MT-TopLap model updated with new experimental DMS with AF3's predicted SARS-CoV-2 RBD-ACE2 complex. In this work, MT-TopLap model is validated with four SARS-CoV-2 RBD-ACE2 datasets from Table S1.

For each of the four datasets, a 10-fold cross-validation is performed to validate AF3-assisted MT-TopLap after pre-training. Metrics such as Pearson correlation coefficient (PCC) and the root mean squared error (RMSE) are used to measure the performance of the pre-trained MT-TopLap model. For an x variable and a y variable, R_p is defined as:

$$\text{PCC} = \frac{\sum(x_i - \bar{x})(y_i - \bar{y})}{\sqrt{\sum(x_i - \bar{x})^2 \sum(y_i - \bar{y})^2}}.$$

Here, x_i is value from x variable of the ith sample and y_i is the value from y variable of the ith sample. $\bar{x}$ and $\bar{y}$ are the mean value of x and y variables respectively.

The root mean squared error (RMSE) can be calculated as follows:

$$\text{RMSE} = \sqrt{\frac{1}{n}\sum_{i=1}^{n}(y_i - \hat{y}_i)^2},$$

where y_i and $\hat{y}_i$ are the predicted and truth values for the ith sample of y respectively. Table S3 shows the numerical results for the 10-fold cross-validation of the four SARS-CoV-2 RBD-ACE2 datasets.

Dataset		PCC	RMSE
6M0J-RBD-1	PDB	0.868	0.947
	AlphaFold3	0.845	1.024
6M0J-RBD-2	PDB	0.956	0.516
	AlphaFold3	0.955	0.527
7T9L-RBD	PDB	0.975	0.331
	AlphaFold3	0.962	0.405
7XB0-RBD	PDB	0.974	0.309
	AlphaFold3	0.968	0.338

Table S3: The numerical PCCs and RMSEs for the 10-fold crossvalidation of the four SARS-CoV-2 RBD-ACE2 datasets.

1.1 Multitask Deep Learning Algorithm

The persistent Laplacian, auxiliary and pre-trained transformer-based features for four datasets (Table S1) are used for the 10-fold cross-validation test. For each dataset level validation, MT-TopLap is first multitask pre-trained using S8338 dataset and several other DMS datasets. For the HK.3 DMS dataset, AF3-assisted MT-TopLap also undergoes pre-training first. The 10-fold cross-validation of experimental HK.3 DMS data is used to validate the fine-tuning of AF3-assisted MT-TopLap. The results are reported in the main paper.

Predicting the changes in binding free energy caused by RBD mutations in RBD-ACE2 complexes is essential for identifying the RBD mutations that drive the evolution of the SARS-CoV-2 virus. For different dataset validation tasks (Table S1) and the S8338 dataset during the multitask pre-training step, the training data for a kth task is $(\mathbf{X}_j^k, \mathbf{y}_j^k)_j^{N_k}$ where D is the feature size, N_k is the number of samples in kth task and $\mathbf{X}_j^k \in \mathbb{R}^{N_k \times D}$ is a feature vector for kth task. Note that $\mathbf{y}_j^k \in \mathbb{R}^{N_k \times 1}$ is the experimental target with the jth sample of the kth task. In a multitask deep neural network model, the main goal is to simultaneously minimize

$$\text{argmin} \sum_{j=1}^{N_k} L(\mathbf{y}_j^k, f(\mathbf{X}_j^k; \{\mathbf{W}^k, \mathbf{b}^k\})),$$

where L is the loss function, f is a function of $\mathbf{X}_j^k$ parameterized by weight of kth task $\mathbf{W}^k$ and bias of kth task $\mathbf{b}^k$. Here, a typical loss function for regression task would be the mean squared error. For instance, the loss function of a kth task can be written as

$$\text{Loss of } k\text{th Task} = \frac{1}{2} \sum_{j=1}^{N_k} L(\mathbf{X}_j^k, \mathbf{y}_j^k) = \frac{1}{2} \sum_{j=1}^{N_k} (\mathbf{y}_j^k - f(\mathbf{X}_j^k; \{\mathbf{W}^k, \mathbf{b}^k\}))^2.$$

To transfer the learning of AF3-assisted MT-TopLap to predict other DMS data such as the HK.3 DMS data, an additional output channel is added to the output layer, and the model is fine-tuned using

experimental enrichment ratios. To do this, we froze the 2nd and 3rd hidden layers and trained MT-TopLap using the added output channel. For the 10-fold cross-validation, the predictions are obtained and validated with the converted BFE changes upon mutation. Standard optimization techniques for feedforward neural networks, along with dropout procedures, are employed to prevent overfitting. The network's structure, including the number of layers and neurons per layer, is determined through grid searches based on 10-fold cross-validations. Subsequently, the hyperparameters for stochastic gradient descent (SGD) with momentum are set according to the network's structure. The network comprises of 6 layers, each containing 15,000 neurons. For SGD with momentum, the hyperparameters include a momentum of 0.9 and a weight decay of 0. The learning rate is set at 0.0001, with a batch size of 50. The DMS datasets undergo pretraining for 500 epochs, followed by 200 epochs for validation and fine-tuning.

1.2 Assessment of AF3-assisted MT-TopLap's performance beyond binding interface

As mentioned in the main paper, AF3-assisted MT-TopLap generated a good performance in predicting the BFE changes upon mutations in support, core and rim regions. These three regions are categorizations of the residues in the PPI's binding interface and are determined based on the rASA values of the monomer and complex (see Results section). To further examine AF3-assisted MT-TopLap capabilities, we analyse the predicted BFE changes upon mutations in the surface and interior regions. The analysis is performed in the Discussion section based on Figure S2. Essentially, AF3-assisted MT-TopLap still performs well in predicting BFE change upon interior mutations but its performance drops when predicting surface mutation-induced BFE changes. During the finetuning of AF3-assisted MT-TopLap using the HK.3 DMS data, the predictions for surface mutations drop to a PCC of 0.557.

2 Feature Generation Process

2.1 ESM-2 Transformer Features

Recent advances in protein property modeling have been driven by large language models trained on extensive datasets of protein sequences. Notable examples include ESM (Evolutionary Scale Modeling) [15] and ProtTrans [16, 17], which leverage deep learning to predict and understand protein structures and functions from their sequences. Hybrid fine-tuning methods, incorporating both local and global evolutionary data, have further enhanced these models' performance. Local evolutionary data captures mutations and variations within closely related species, while global data encompasses a wider range of species and evolutionary distances. This combination improves the models' generalization and accuracy in predicting protein behavior.

The ESM model can also be fine-tuned using data from specific downstream tasks or local multiple sequence alignments, making it adaptable to various applications. This flexibility is particularly useful for capturing subtle sequence variations crucial for tasks like predicting protein-protein interactions or enzyme functions.

In our research, we utilized the ESM-2 transformer, trained on a dataset of 250 million sequences using a masked filling procedure. This method involves randomly masking portions of the input sequence and training the model to predict the missing parts, allowing it to learn complex sequence patterns and

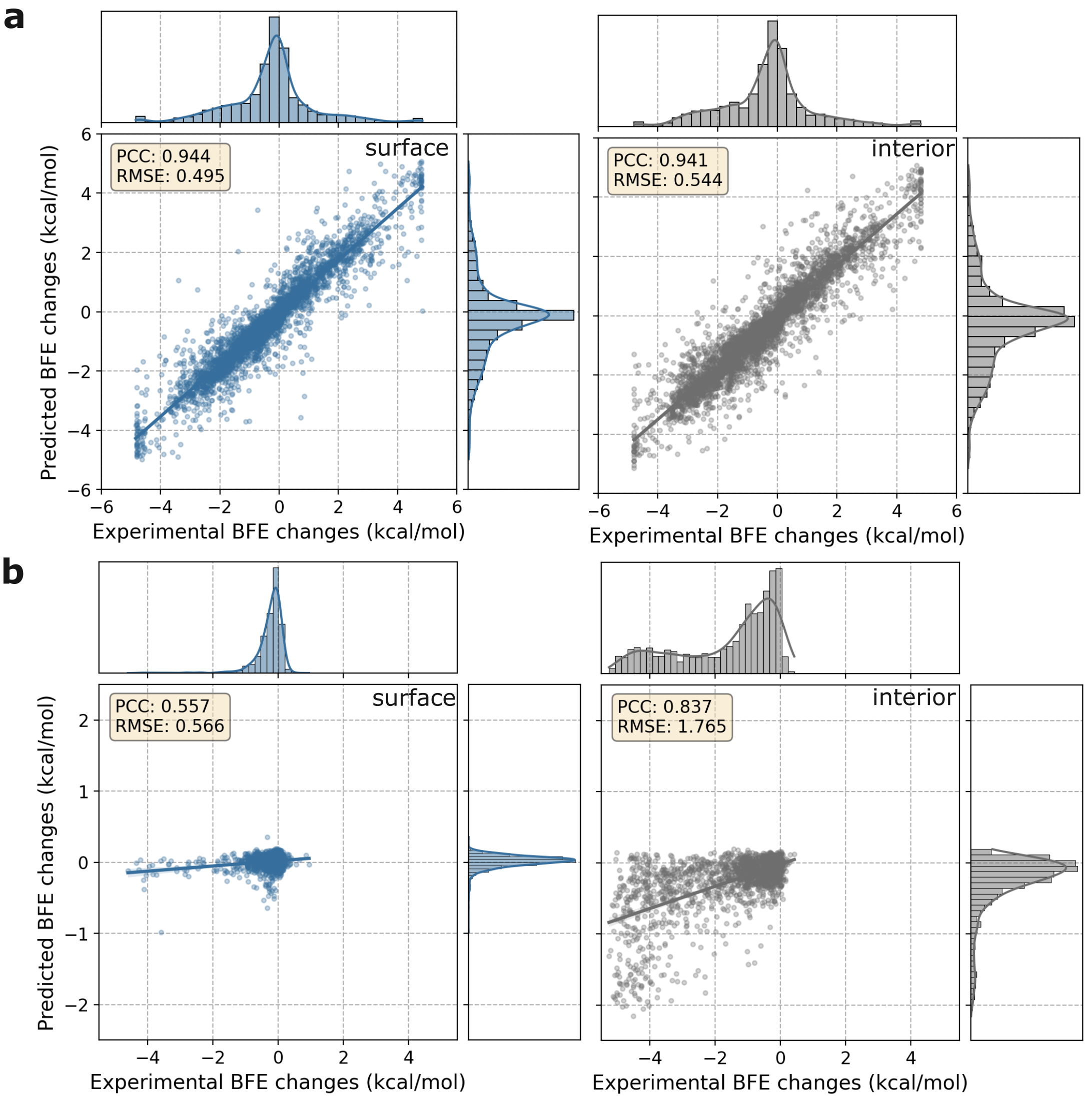

Figure S2: (a): AF3-assisted MT-TopLap's 10-fold cross-validations for the four SARS-CoV-2 RBD-ACE2 datasets show an average PCC of 0.933 and the average RMSE of 0.570 (see Figure 1(a)-(b)). The combined prediction results for surface and interior region types all four datasets according to Figure 2(a) with PCCs of 0.944 and 0.941. The converted BFE changes and MT-TopLap's predictions are compared. (b): The 10-fold cross-validations of deep mutational scanning on the HK.3 DMS data[14] shows a PCC of 0.81 (see Figure 1(c)). Prediction results for surface and interior region types according to Figure 2(a) with PCCs of 0.557 and 0.837 respectively. The converted BFE changes and MT-TopLap's predictions are compared.

dependencies. The ESM-2 transformer features a 34-layer architecture with 650 million parameters, making it exceptionally powerful in this domain.

The main role of the ESM transformer in our work was to generate sequence embeddings. Each layer of the model encoded a sequence of length L into a 1,280× L matrix, excluding the start and end tokens. For our study, we used the sequence representation from the final (34th) layer, averaging along the sequence length to produce a 1,280-component vector. This process allowed us to leverage the deep contextual information captured by the ESM-2 transformer, enabling detailed analysis and predictions. These high-dimensional embeddings provided a compact and informative representation of protein sequences, essential for accurate modeling and various downstream analyses like protein structure prediction, functional annotation, and interaction mapping.

2.2 Auxiliary descriptors

In our MT-TopLap model, the persistent Laplacian descriptors incorporate structural data and biomolecular interactions as topological and geometrical attributes. To take into account the influence of essential chemical and physical properties, we incorporate several additional features calculated from the chemical and physical information extracted from their structural data. These additional features are primarily divided into two categories: features at the atom level and features at the residue level.

Atom-level features Features at the atom level are derived from the individual atoms within the PPI structures. These features include atomic types, partial charges, and van der Waals radii. By incorporating these properties, we can capture the nuances of atomic interactions, such as hydrogen bonding, ionic interactions, and hydrophobic effects. Atom-level features also consider the spatial arrangement and distances between atoms, allowing for a detailed analysis of molecular geometry and interaction patterns. In terms of atom types, atoms are divided into seven groups specific to elements, namely, C, N, O, S, H, all heavy atoms, and all atoms. Based on the distance of atoms from the mutation site, atoms are further divided into three groups: atoms at the mutation site, atoms near the mutation site (within 10Å from the mutation site), and all atoms. Finally, we consider three scenarios: the wild type, the mutant type, and their difference, respectively.

One of the key atom-level features is the MIBPB features. Our in-house MIBPB (Molecular Interaction-based Potential of Mean Force and the Poisson-Boltzmann) software[18] calculates the electrostatic solvation free energy for each atom using the Poisson-Boltzmann model. MIBPB incorporate both the potential of mean force and Poisson-Boltzmann electrostatics to characterize biomolecular interactions. MIBPB considers the dielectric properties of both the biomolecule and the solvent, discretizing the biomolecule's surface into a grid, and calculating the electrostatic potential. The solvation free energy is then computed by summing the contributions from all atoms, which provides insights into molecular interactions and stability, resulting in the generation of 63 features. Note that for our MIBPB input, we apply the PDB2PQR software [19] and a AMBER/CHARMM27 force field is used to calculate the radius and partial charge for each atom stored in a pqr file. In short, MIBPB offers a comprehensive view of the molecular interaction landscape, capturing both energetic and electrostatic contributions to protein behavior. By integrating MIBPB features with persistent Laplacian descriptors, atom-level, and residue-level features, the MT-TopLap model achieves a robust and holistic representation of the PPI interactions.

Apart from the MIBPB features, we also incorporate the partial charge features. The partial charge features consists of the total of the partial charges and the total of the absolute values of the partial charges for each atomic group. In total, 126 features are generated.

Also, we calculate the Coulomb energy of a single atomby summing the pairwise Coulomb energy with all other atoms, using the formula:

$$C_i = \sum_{j,j\neq i} k_e \frac{q_i q_j}{r_{ij}}.$$

Here, k_e is the Coulomb's constant, which is set to 1 for all calculations. We only consider five types of elements (C, N, O, S, and all heavy atoms) when creating Coulomb interaction features. We calculate both the Coulomb interaction energy and its absolute value. This results in a total of 90 features.

Other atom-level features include the van der Waals energy and atom-level solvent excluded surface areas. The atom-level solvent excluded surface areas is calculated using the ESES software[20]. The areas of all atoms belonging to the same group are summed up to create a single feature. In total, 7*3*3 = 63 features are generated. The van der Waals energy of an ith atom can be calculated by adding up the pairwise Lennard-Jones potentials with all other atoms. This calculation is only considered for five types of elements: C, N, O, S, and all heavy atoms.

$$V_i = \sum_{j,j\neq i} \epsilon \left[\left(\frac{r_i + r_j}{r_{ij}} \right)^{12} - 2 \left(\frac{r_i + r_j}{r_{ij}} \right)^{6} \right].$$

Here, ϵ is the depth of the potential well. $\epsilon = 1$ is also used in this calculation. All van der Waals interactions are computed for each atom by comparing against all heavy atoms with a cutoff of 40Å. 45 features are generated.

Residue-level features The residue-level features are broken down into several key components. The first key component is the mutation site neighborhood amino acid composition. For this component, residues within a 10Å radius of the mutation site are considered part of the neighborhood. The distances between residues are calculated using their C_α atoms. To capture the local environment, amino acid residues are categorized into five groups: hydrophobic, polar, positively charged, negatively charged, and special cases. Each of these groups has distinct chemical properties that influence the protein's behavior and interactions.

- Hydrophobic Residues: These are amino acids that avoid water and tend to cluster together within the protein core, contributing to its stability. Common hydrophobic residues include alanine, valine, leucine, and isoleucine.

- Polar Residues: These amino acids can form hydrogen bonds and interact with water, influencing the protein's solubility and interaction with other molecules. Examples include serine, threonine, and asparagine.

- Positively Charged Residues: These amino acids have a positive charge at physiological pH and can form ionic bonds with negatively charged residues or molecules. Examples include lysine, arginine, and histidine.

- Negatively Charged Residues: These amino acids have a negative charge at physiological pH and can form ionic bonds with positively charged residues or molecules. Examples include aspartic acid and glutamic acid.
- Special Cases: This group includes amino acids with unique properties, such as proline, which induces kinks in protein chains, and cysteine, which can form disulfide bonds that stabilize protein structure.

For each group, we calculate both the count and percentage of amino acids in the neighboring sites. This provides a detailed profile of the local environment, resulting in ten features (five groups multiplied by two metrics). Additionally, we generate features based on the sum, average, and variance of residue volumes, surface areas, weights, and hydropathy scores. Hydropathy scores indicate the hydrophobic or hydrophilic nature of the residues. Summing these metrics gives twelve features (three statistics multiplied by four attributes). Altogether, this approach yields a total of 22 features, offering a comprehensive view of the mutation site neighborhood's composition and characteristics.

Next are the pK_a shifts. The pK_a values of seven ionizable amino acids—ASP, GLU, ARG, LYS, HIS, CYS, and TYR—are computed using the PROPKA software [21]. These values reflect the propensity of amino acid side chains to ionize, which affects their charge states under different pH conditions. Changes in pK_a values between the wild type and its mutated version, known as pK_a shifts, can significantly impact the protein's function and interactions.

We calculate various statistics of these pK_a shifts:

- Maximum and Minimum pK_a Shifts: These values highlight the most extreme changes in ionization propensity, which could indicate major alterations in protein behavior.
- Sum of pK_a Shifts: This provides an overall measure of the magnitude of changes across all ionizable residues.
- Sum of Absolute Values of pK_a Shifts: This statistic emphasizes the total extent of ionization changes, regardless of direction.
- Minimum of Absolute Values of pK_a Shifts: This helps identify the least altered ionizable residues, which might remain stable post-mutation.

In addition, we compute the sum and absolute sum of pK_a shifts for each of the seven ionizable amino acid groups, resulting in fourteen additional features. Collectively, this results in a total of nineteen features that provide insights into how mutations affect the protein's ionization properties and, consequently, its interactions and stability.

Lastly, we consider the secondary structure information. Using the SPIDER2 software [22], we analyze the secondary structure tendencies of residues at the mutation site. Secondary structures, such as coils, helices, and strands, are fundamental elements of protein architecture and directly influence its function. SPIDER2 provides probability scores for residues to adopt coil, helix, or strand structures. These scores are indicative of the structural tendencies and flexibility of the PPI at the mutation site. In addition to probability scores, torsion angles, which describe the rotation of the backbone around bonds, are also calculated. For each residue, we consider these metrics for both the wild type and the mutated type, and their differences, to capture the mutation's impact on secondary structure. This results in twelve

features (four metrics multiplied by three categories), offering a detailed understanding of how mutations might alter the PPI's secondary structure. By integrating these comprehensive residue-type features, the MT-TopLap model gains a nuanced understanding of the local environment, pH shifts, and secondary structural changes around mutation sites. This holistic approach enhances the model's ability to predict and analyze PPI behavior and interactions with high accuracy.

2.3 Harmonic Spectral Features

Persistent homology (PH) is a key component in understanding the harmonic spectra of Persistent Laplacians (PL). PH uses homology groups to reveal the persistence of topological invariants, providing harmonic spectral information within the PL framework. The process of generating site and element-specific PH features follow a methodology similar to that of PL, employing analogous filtration construction.

In zero dimensions, the filtration parameter is divided into several equally spaced bins: [0, 0.5], (0.5, 1], continuing up to (5.5, 6]Å. Within each bin, the death values of the bars are summed, resulting in a matrix of features. Specifically, we calculate 12×18 features, where 12 represents the number of bins and 18 is derived from the variety of atomic pairs or elements being considered. For each bin, we tally the number of persistent bars, creating a nine-dimensional vector for each point cloud. This detailed counting process is carried out for each of the nine single atomic pairs, resulting in a total of 216 PH features for a protein in zero dimensions.

For one and two dimensions, we apply the same statistical featurization to persistent bars within PH. Filtration in these dimensions employs the Alpha complex with the DE distance. The Alpha complex is a subcomplex of the Delaunay triangulation, constructed based on the proximity of points within a given distance, effectively capturing the geometric structure of the protein. Due to the limited number of atoms in local protein structures, which form only a few high-dimensional simplexes, the focus remains on the harmonic spectra of persistent Laplacians. This captures essential topological invariants of high-dimensional interactions, providing a nuanced and detailed view of the molecular structure.

Using the GUDHI (Geometry Understanding in Higher Dimensions) library[23], we represent the persistence of harmonic spectra through persistent barcodes. These barcodes visually and quantitatively capture the birth and death of topological features across different scales. We generate topological feature vectors by calculating statistical measures of bar lengths, births, and deaths. Bars shorter than 0.1Å are excluded due to their lack of clear physical significance. The calculated statistics include the sum, maximum, and mean of bar lengths; the minimum and maximum of bar birth values; and the minimum and maximum of bar death values. Each set of point clouds results in a seven-dimensional vector, with these features computed for nine single atomic pairs and one heavy atom pair. This yields a total of 140 features for one- and two-dimensional PL vectors of a protein.

To create a comprehensive PH embedding, we combine features across different dimensions. We concatenate vectors for the wild type, mutant, and their differences, resulting in a feature vector of length 648. This thorough and multi-dimensional approach ensures that both the local and global topological characteristics of protein-protein interactions (PPI) are captured, allowing for more precise modeling and analysis of PPI binding behavior and biomolecular interactions.

2.4 Software and resources

Here, we indicate all the softwares and resources used in our feature generation process.

To produce mutant proteins, the Jackal software [24] is utilized. The MT-TopLap model itself is constructed using PyTorch [25], a flexible and efficient deep learning library. For generating PQR files, which contain the partial charge data of the proteins, the PDB2PQR software [26] is employed. These PQR files for both wild-type and mutant proteins are generated using the AMBER force field, ensuring accurate representation of the molecular charges.

The ESES in-house online software package [27] and the MIBPB tool [18] are used to calculate solvation energy and surface area information. These calculations are crucial for understanding the interactions and stability of the proteins in their respective environments. The pK_a values of amino acids are computed using the PROPKA software package [28], which predicts pK_a shifts that can affect protein function.

Position-specific scoring matrices (PSSM) are constructed using the ncbi-blast software [29] with an nr database, providing insights into the evolutionary conservation of protein sequences. For secondary structure analysis and sequence-based torsion angle information, the SPIDER2 software [30] is utilized, offering detailed predictions of protein folding patterns.

Finally, the GUDHI software library [23] is employed to calculate persistent Laplacian descriptors for both Vietoris-Rips (VR) complexes and alpha complexes. These descriptors provide topological insights into the protein structures, enhancing the predictive capabilities of the MT-TopLap model.

This comprehensive suite of tools and software packages ensures the MT-TopLap model is equipped with robust and accurate data for analyzing protein interactions and predicting their behavior.

References

[1] J. Wee, J. Chen, and G.-W. Wei, "Preventing future zoonosis: SARS-CoV-2 mutations enhance human–animal cross-transmission," *Computers in Biology and Medicine*, vol. 182, p. 109101, 2024.

[2] J. Chen, D. R. Woldring, F. Huang, X. Huang, and G.-W. Wei, "Topological deep learning based deep mutational scanning," *Computers in Biology and Medicine*, vol. 164, p. 107258, 2023.

[3] T. N. Starr, A. J. Greaney, S. K. Hilton, D. Ellis, K. H. Crawford, A. S. Dingens, M. J. Navarro, J. E. Bowen, M. A. Tortorici, A. C. Walls, *et al.*, "Deep mutational scanning of SARS-CoV-2 receptor binding domain reveals constraints on folding and ACE2 binding," *Cell*, vol. 182, no. 5, pp. 1295–1310, 2020.

[4] T. W. Linsky, R. Vergara, N. Codina, J. W. Nelson, M. J. Walker, W. Su, C. O. Barnes, T.-Y. Hsiang, K. Esser-Nobis, K. Yu, *et al.*, "De novo design of potent and resilient hACE2 decoys to neutralize SARS-CoV-2," *Science*, vol. 370, no. 6521, pp. 1208–1214, 2020.

[5] J. Lan, J. Ge, J. Yu, S. Shan, H. Zhou, S. Fan, Q. Zhang, X. Shi, Q. Wang, L. Zhang, *et al.*, "Structure of the SARS-CoV-2 spike receptor-binding domain bound to the ACE2 receptor," *nature*, vol. 581, no. 7807, pp. 215–220, 2020.

[6] K. K. Chan, D. Dorosky, P. Sharma, S. A. Abbasi, J. M. Dye, D. M. Kranz, A. S. Herbert, and E. Procko, "Engineering human ACE2 to optimize binding to the spike protein of SARS coronavirus 2," *Science*, vol. 369, no. 6508, pp. 1261–1265, 2020.

[7] T. N. Starr, A. J. Greaney, C. M. Stewart, A. C. Walls, W. W. Hannon, D. Veesler, and J. D. Bloom, "Deep mutational scans for ACE2 binding, RBD expression, and antibody escape in the SARS-CoV-2 Omicron BA. 1 and BA. 2 receptor-binding domains," *PLoS pathogens*, vol. 18, no. 11, p. e1010951, 2022.

[8] D. Mannar, J. W. Saville, X. Zhu, S. S. Srivastava, A. M. Berezuk, K. S. Tuttle, A. C. Marquez, I. Sekirov, and S. Subramaniam, "SARS-CoV-2 Omicron variant: Antibody evasion and cryo-EM structure of spike protein–ACE2 complex," *Science*, vol. 375, no. 6582, pp. 760–764, 2022.

[9] L. Li, H. Liao, Y. Meng, W. Li, P. Han, K. Liu, Q. Wang, D. Li, Y. Zhang, L. Wang, *et al.*, "Structural basis of human ACE2 higher binding affinity to currently circulating Omicron SARS-CoV-2 sub-variants BA. 2 and BA. 1.1," *Cell*, vol. 185, no. 16, pp. 2952–2960, 2022.

[10] J. Jankauskaitė, B. Jiménez-García, J. Dapkūnas, J. Fernández-Recio, and I. H. Moal, "SKEMPI 2.0: an updated benchmark of changes in protein–protein binding energy, kinetics and thermodynamics upon mutation," *Bioinformatics*, vol. 35, no. 3, pp. 462–469, 2019.

[11] E. F. Pettersen, T. D. Goddard, C. C. Huang, E. C. Meng, G. S. Couch, T. I. Croll, J. H. Morris, and T. E. Ferrin, "UCSF ChimeraX: Structure visualization for researchers, educators, and developers," *Protein Science*, vol. 30, no. 1, pp. 70–82, 2021.

[12] J. Wee and G.-W. Wei, "Evaluation of AlphaFold 3's Protein–Protein Complexes for Predicting Binding Free Energy Changes upon Mutation," *Journal of Chemical Information and Modeling*, vol. 64, no. 16, pp. 6676–6683, 2024.

[13] J. Chen, Y. Qiu, R. Wang, and G.-W. Wei, "Persistent Laplacian projected Omicron BA. 4 and BA. 5 to become new dominating variants," *Computers in Biology and Medicine*, vol. 151, p. 106262, 2022.

[14] A. L. Taylor and T. N. Starr, "Deep mutational scanning of SARS-CoV-2 Omicron BA. 2.86 and epistatic emergence of the KP. 3 variant," *Virus Evolution*, vol. 10, no. 1, p. veae067, 2024.

[15] A. Rives, J. Meier, T. Sercu, S. Goyal, Z. Lin, J. Liu, D. Guo, M. Ott, C. L. Zitnick, J. Ma, *et al.*, "Biological structure and function emerge from scaling unsupervised learning to 250 million protein sequences," *Proceedings of the National Academy of Sciences*, vol. 118, no. 15, p. e2016239118, 2021.

[16] A. Vaswani, N. Shazeer, N. Parmar, J. Uszkoreit, L. Jones, A. N. Gomez, Ł. Kaiser, and I. Polosukhin, "Attention is all you need," *Advances in Neural Information Processing Systems*, vol. 30, 2017.

[17] J. Devlin, M.-W. Chang, K. Lee, and K. Toutanova, "BERT: Pre-training of Deep Bidirectional Transformers for Language Understanding," in *North American Chapter of the Association for Computational Linguistics*, 2019.

[18] D. Chen, Z. Chen, C. Chen, W. Geng, and G.-W. Wei, "Mibpb: a software package for electrostatic analysis," *Journal of computational chemistry*, vol. 32, no. 4, pp. 756–770, 2011.

[19] T. J. Dolinsky, P. Czodrowski, H. Li, J. E. Nielsen, J. H. Jensen, G. Klebe, and N. A. Baker, "PDB2PQR: Expanding and upgrading automated preparation of biomolecular structures for molecular simulations," *Nucleic Acids Res*, vol. 35, pp. W522–525, 2007.

[20] B. Liu, B. Wang, R. Zhao, Y. Tong, and G. W. Wei, "ESES: software for Eulerian solvent excluded surface," *Preprint*, 2015.

[21] D. C. Bas, D. M. Rogers, and J. H. Jensen, "Very fast prediction and rationalization of pKa values for protein–ligand complexes," *Proteins: Structure, Function, and Bioinformatics*, vol. 73, no. 3, pp. 765–783, 2008.

[22] Y. Yang, R. Heffernan, K. Paliwal, J. Lyons, A. Dehzangi, A. Sharma, J. Wang, A. Sattar, and Y. Zhou, "SPIDER2: A package to predict secondary structure, accessible surface area, and main-chain torsional angles by deep neural networks," *Prediction of protein secondary structure*, pp. 55–63, 2017.

[23] C. Maria, J.-D. Boissonnat, M. Glisse, and M. Yvinec, "The GUDHI library: Simplicial complexes and persistent homology," in *Mathematical Software–ICMS 2014: 4th International Congress, Seoul, South Korea, August 5-9, 2014. Proceedings 4*, pp. 167–174, Springer, 2014.

[24] J. Z. Xiang and B. Honig, "Jackal: A protein structure modeling package," *Columbia University and Howard Hughes Medical Institute, New York*, 2002.

[25] A. Paszke, S. Gross, F. Massa, A. Lerer, J. Bradbury, G. Chanan, T. Killeen, Z. Lin, N. Gimelshein, L. Antiga, *et al.*, "Pytorch: An imperative style, high-performance deep learning library," *Advances in neural information processing systems*, vol. 32, 2019.

[26] T. J. Dolinsky, J. E. Nielsen, J. A. McCammon, and N. A. Baker, "PDB2PQR: An automated pipeline for the setup of Poisson–Boltzmann electrostatics calculations," *Nucleic acids research*, vol. 32, no. suppl_2, pp. W665–W667, 2004.

[27] B. Liu, B. Wang, R. Zhao, Y. Tong, and G.-W. Wei, "ESES: Software for Eulerian solvent excluded surface," 2017.

[28] H. Li, A. D. Robertson, and J. H. Jensen, "Very fast empirical prediction and rationalization of protein pKa values," *Proteins: Structure, Function, and Bioinformatics*, vol. 61, no. 4, pp. 704–721, 2005.

[29] M. Johnson, I. Zaretskaya, Y. Raytselis, Y. Merezhuk, S. McGinnis, and T. L. Madden, "NCBI BLAST: A better web interface," *Nucleic acids research*, vol. 36, no. suppl_2, pp. W5–W9, 2008.

[30] R. Heffernan, K. Paliwal, J. Lyons, A. Dehzangi, A. Sharma, J. Wang, A. Sattar, Y. Yang, and Y. Zhou, "Improving prediction of secondary structure, local backbone angles and solvent accessible surface area of proteins by iterative deep learning," *Scientific reports*, vol. 5, no. 1, p. 11476, 2015.
